# Energy-Aware RFID Anti-Collision Protocol

**DOI:** 10.3390/s18061904

**Published:** 2018-06-11

**Authors:** Laura Arjona, Hugo Landaluce Simon, Asier Perallos Ruiz

**Affiliations:** Faculty of Engineering, University of Deusto and DeustoTech-Fundacion Deusto, Deusto Foundation, 48007 Bilbao, Spain; hlandaluce@deusto.es (H.L.S.); perallos@deusto.es (A.P.R.)

**Keywords:** radio frequency identification, EPC-global standard, anti-collision, tag estimation, energy-aware

## Abstract

The growing interest in mobile devices is transforming wireless identification technologies. Mobile and battery-powered Radio Frequency Identification (RFID) readers, such as hand readers and smart phones, are are becoming increasingly attractive. These RFID readers require energy-efficient anti-collision protocols to minimize the tag collisions and to expand the reader’s battery life. Furthermore, there is an increasing interest in RFID sensor networks with a growing number of RFID sensor tags. Thus, RFID application developers must be mindful of tag anti-collision protocols. Energy-efficient protocols involve a low reader energy consumption per tag. This work presents a thorough study of the reader energy consumption per tag and analyzes the main factor that affects this metric: the frame size update strategy. Using the conclusion of this analysis, the anti-collision protocol Energy-Aware Slotted Aloha (EASA) is presented to decrease the energy consumption per tag. The frame size update strategy of EASA is configured to minimize the energy consumption per tag. As a result, EASA presents an energy-aware frame. The performance of the proposed protocol is evaluated and compared with several state of the art Aloha-based anti-collision protocols based on the current RFID standard. Simulation results show that EASA, with an average of 15 mJ consumed per tag identified, achieves a 6% average improvement in the energy consumption per tag in relation to the strategies of the comparison.

## 1. Introduction

Radio Frequency Identification (RFID) technology is becoming increasingly popular to the point where almost anything can be tagged. This is mainly because the cost of commercial RFID tags is negligible compared to the value of the products to which they are attached. Current examples of RFID expansion can be found in sensing, activity recognition and localization systems [1,2].

RFID technology uses a spectrum of radio frequency to transfer the identification information between two communication devices: reader and tags [3]. The coexistence of several tags provides RFID technology with a great flexibility at the expense of the tag collision problem. Tags share the same communication channel (the air) and may respond simultaneously to the same interrogation command, interfering and garbling their waveforms. The reader then is unable to interpret the information received from the tags, requiring a re-transmission and extending the tag identification time. Anti-collision protocols are then proposed to arbitrate tags’ responses and to increase the number of tags identified by a time unit.

In the literature, three main types of anti-collision protocols have been reported: Aloha-based, tree-based and hybrid protocols. The three types of protocols can be applied to active (battery-operated tags), passive (tags backscatter information) or semi-passive (combination of active and passive) RFID systems. Tree-based protocols [4,5,6], in essence, split colliding tags into subsets and further split the subsets repeatedly up to the successful response of all the tags that are within the interrogation zone. Aloha-based protocols [7] divide time into frames so that tags randomly choose one slot per frame to respond. While in Frame-Slotted Aloha (FSA), the frame size *L* is fixed during the identification process, in Dynamic Frame Slotted Aloha (DFSA), it is variable, and the protocol’s performance is greatly influenced by the update of *L*. The fact that the standard EPC-global Class-1 Generation-2 (EPC C1G2) [8] currently uses a DFSA structure to arbitrate collisions highlights the research relevance of this scheme. For this reason, this work focuses on DFSA protocols. Finally, hybrid protocols combine the advantages of tree and Aloha protocols [9].

RFID systems, which have already been adopted in applications such as supply chains, are now considered a front-runner of the ubiquitous era with the emergence of mobile RFID [10]. Currently, mobile and battery-powered RFID readers, such as hand readers and smart phones, are increasingly being used [11,12]. Most popular applications focus on RFID-based indoor localization and identification systems with mobile readers [13,14,15]. Therefore, it is desired to expand the reader’s battery life by using an energy-aware tag anti-collision protocol. This work presents the Energy-Aware Slotted Aloha (EASA) anti-collision protocol.

Defining *n* as the the total number of tags inside the reader interrogation zone and defining *E* as the total amount of energy consumed by the reader to identify the *n* tags, the metric *E*/*n* is defined as the energy per tag identified consumed by the reader, in one identification round. The ratio *E*/*n* provides information not only about the energy consumption of the RFID system using a particular anti-collision protocol, but it also provides information about the scalability of the system. Ideally, if the system is scalable to large population sizes, the ratio *E*/*n* should not vary considerably with increasing *n*, meaning that the reader energy consumption per tag is approximately constant and independent of *n*. In previous research, most energy saving protocols aim to reduce the energy cost of the reader and tags separately for active RFID systems [16,17,18,19]. They are not suitable for passive RFID systems. Other recent works focus on passive RFID systems, but they use tree-based anti-collision protocols [4,11,20].

The proposed protocol updates the frame size so that E/n is minimized. To do so, the frame size is set as a function of the estimated tag set size and the parameter ρ. The parameter ρ is defined as the ratio between the tag set size *n* and the frame size *L*, and its value is updated with the characteristics of the physical RFID system. The results of the performance evaluation show that the proposed protocol decreases E/n in relation to the strategies in the comparison. The following main contributions are made in this work:

1. Analytical study of the *L* that minimizes the E/n metric.
2. Presentation of a novel anti-collision protocol: EASA; the proposed protocol applies the results obtained in the previous contribution to decrease E/n in an RFID system based on EPC C1G2.
3. E/n evaluation of EASA and comparison with several anti-collision protocols of the state of the art.

The rest of the paper is organized as follows. Section 2 presents the RFID Standard EPC C1G2 and several related Aloha-based anti-collision protocols in the literature. Section 3 provides a thorough analysis of the main factor that affects E/n and obtains the value of *L* that minimizes it. The proposed EASA anti-collision protocol is presented in Section 4. Section 5 provides the results of the performance evaluation followed by the study of the physical identified limitations and future work in Section 6. Finally, Section 6 concludes this paper.

## 2. Background

Some definitions are provided to properly set the background of this work and to better understand the main contributions: Bulleted lists look like this:

- A command is a bit-string transmitted by the reader to the tags.
- An inventory round is the period of time that begins when the reader transmits the initial command (Qc), and it ends when the reader interrupts the identification process and the tags loose their state. Ideally, an inventory round ends when all the tags in the reader interrogation zone have been identified.
- A slot is a period of time that separate tags’ responses. Conventionally, three types of slots are considered attending to the tags’ responses to the reader’s commands: idle (none of the tags reply), single (only one tag replies) and collision (more than one tag replies in the same slot). The duration of each type of slot is referred as $T_{i}$, $T_{s}$ and $T_{k}$, respectively. These slots are accurately specified in the current standard [8], and their duration is determined by the link timing parameters $\left(T_{1},T_{2},T_{3}\right)$.
- A frame is a sequence of slots. Tags can respond in only one slot per frame. An identification process is composed of a set of frames.

**Figure 1:**
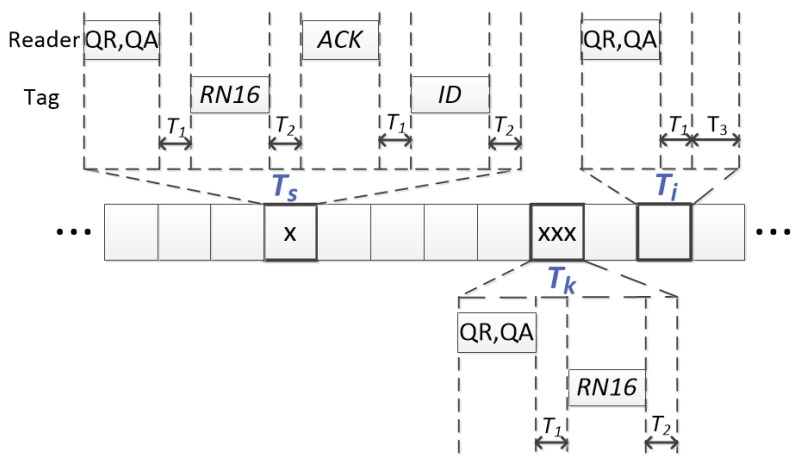
Link timing of EPC C1G2.

The strategies analyzed in this work are based on the RFID transmission model between the reader and the tags defined in Figure 1, meeting EPC C1G2 requirements [8]. In this figure, three types of slots are represented: a single slot, with only one tag response (x), a collision slot, with three tags’ simultaneous responses (xxx) and an idle slot, with no response. According to this figure, a single slot has a duration $T_{s}$, and this slot involves two reader commands and two tag’s responses. A collision slot has a duration $T_{k}$, and it involves one reader command and one tags’ response. Finally, an idle slot has a duration $T_{i}$, and it only involves one reader command. The parameter $T_{1}$ refers to the time needed for the tags to generate their responses after every reader command. The parameter $T_{2}$ refers to the time needed for the reader to receive all the tag transmissions. Finally, a slot will be considered idle when the reader waits for the tags’ responses for a time $T_{3}$.

Now that the main concepts have been explained, the current standard in the RFID system is presented.

### 2.1. RFID Standard EPC C1G2

EPC C1G2 [8], the current standard in RFID systems, defines the requirements followed by the proposed protocol in this work. EPC C1G2 employs a DFSA protocol to arbitrate collisions, known as the Slot Counter protocol. The probability of collision is sensitive to the choice of *L*, which is dynamically updated by means of the parameter named *Q* (*L* = $2^{Q}$). The optimum *L* setting depends on the unknown number of responding tags.

The slot counter protocol schedules tags’ responses along time slots. In order to manage the identification process, the reader begins with transmitting Qc once and then alternates between the QueryAdjust(QA) and QueryRep(QR) commands. QA starts a new frame with the updated size and arranges that the tags randomly select a slot in the frame (the initial value of their internal slot counter SC), while QR tells the tags to decrement SC. Thus, when SC = 0, the tag transmits a 16-bit random number (RN16); and once it is acknowledged (ACK), the tag transmits its EPC code of length *k*.

### 2.2. Background of DFSA Anti-Collision Protocols

Currently, the EPC C1G2 standard is followed by most RFID manufacturers, enhancing the research relevance of DFSA-based protocols. Consequently, many DFSA protocols based on the standard have recently appeared with the main objective of improving a set of metrics regarding the process of tag identification. Several DFSA protocols can be found in the literature that update *L* using the tag set size estimated by the reader, referred to as $\hat{n}$.

Prior to presenting the most relevant single-reader strategies, a system model with one reader and *n* tags is defined. A DFSA frame of size *L* is defined. The variables $c_{s}$, $c_{k}$ and $c_{i}$ correspond to the number of single, collision and idle slots in the frame, respectively, and up to the current slot. Additionally, $p_{s}$, $p_{k}$ and $p_{i}$ correspond to the probability that only one tag, no tag or more than one tag occupies a slot, respectively. The examination slot refers to the particular slot within each frame where *L* is updated. Some of the most relevant DFSA-based protocols in the literature are introduced next.

#### 2.2.1. Eom

Eom et al. [21] introduced a DFSA anti-collision protocol that updates *L* according to the estimated tag set size. The estimation algorithm is based on the number of collided tags per slot, referred to as γ. On the one hand, the protocol proposed by the authors in [21] shows a positive performance in terms of the estimation error and the total number of slots used for identification. On the other hand, the authors did not distinguish between the three types of slots to measure the total number of slots. Thus, the comparison with the rest of the protocols is unfair.

#### 2.2.2. ILCM-FbF

The protocol Improved Linearized Combinational Model with Frame by Frame examination of *L* (ILCM-FbF) for the optimal frame size adaptation was introduced by Solic et al. in [7]. The authors presented a DFSA protocol based on the estimation of the tag population with a linear function that depends on $c_{k}$ and *L*. Then, at the end of the frame, *L* is updated using the value of $\hat{n}$. The simulation scenario is limited, because the results are only compared with the slot counter protocol.

#### 2.2.3. ILCM-SbS

The protocol Improved Linearized Combinational Model with Slot by Slot examination of *L* (ILCM-SbS) was presented by Solic et al. in [22] as an improved version of ILCM-FbF [7]. Simulation results showed that ILCM-SbS lowers the time required to identify a set of tags compared with some protocols of the state of the art. However, this strategy might overload a reader that has only a limited capacity, because *L* is calculated at every slot.

#### 2.2.4. Chen14

Chen [23] presented an anti-collision protocol (Chen14) that examines *L* at just one slot per frame, determined as L/i, claiming to significantly reduce the number of total examination slots. The presented protocol updates *L* as a function of $\hat{n}$, and then, *L* is updated based on $\hat{n}$. Simulation results showed an improved performance in terms of normalized throughput, defined as $Throughput=c_{s}/\left(c_{s}+c_{k}+c_{i}\right)$. However, this metric assumes equal duration for each type of slot, and contrasting the EPC C1G2 requirements, these slots have different durations.

#### 2.2.5. Chen16

Chen proposed in [24] an anti-collision algorithm (Chen16) based on the early and optimal adjustment of the frame length with the aim of maximizing the normalized throughput (*U*), defined as $U=\left(c_{s}T_{s}\right)/\left(c_{s}T_{s}+c_{i}T_{i}+c_{k}T_{k}\right)$. This protocol was presented as an extension of the study in [23]. In this Chen16 protocol, the tag set size is estimated in every frame at the examination point L/5. The value of this slot has been selected as the slot where maximum *U* is obtained. Based on the previous $\hat{n}$, if a new frame is required, the author updates *L* with the variable *y*, where *y* is expressed as a second-order polynomial.

Simulation results show competitive values regarding *U*, but the function defined to set *y* is not valid for all the range of $T_{k}$/$T_{i}$. Particularly, if $T_{k}>>T_{i}$, *y* takes negative values, leading to negative values for *L*. Additionally, the examination point L/5 has been set based on a particular scenario with specific timing parameters. Therefore, this value might not be appropriate for a scenario with different timing settings of the RFID system.

#### 2.2.6. SSA and DSSA

The Segment-by-Segment Aloha protocol (SSA) was proposed in [25] to effectively decrease the frame adjustment times with satisfactory throughput. In this protocol, one frame is composed of a set of slot-segments, and each slot-segment is composed of $s_{L}$ continuous time slots, where $s_{L}$ = 4. In order to increase the throughput of SSA, the authors introduced the Dynamic SSA (DSSA) protocol. DSSA varies $s_{L}$ dynamically by tracking, in real time, the number of single slots. Both protocols present a positive performance regarding the throughput and the number of tags identified per second. However, the authors assume a simulation scenario where the protocols are compared with just one additional protocol, and for one specific scenario, with a particular set of timing configuration.

## 3. Energy-Aware Aloha Frame Analysis

Traditionally, the most common metric to evaluate the performance of an RFID anti-collision protocol has been the Slot Efficiency (SE) [26], defined as $SE=c_{s}/\left(c_{i}+c_{s}+c_{k}\right)$. Ideally, an anti-collision protocol is desired to reach SE=1, meaning that just one slot per tag is required for the complete tag set identification. However, this is not achievable in practical applications, where collision and idle slots are present. An anti-collision protocol reaches the maximum SE when the frame size equals the number of tags, that is *L* = *n* [26]. However, this condition only applies when $T_{i}$ = $T_{s}$ = $T_{k}$. The EPC C1G2 standard specifies different durations for idle, single and collision slots, referred to as $T_{i}$, $T_{s}$ and $T_{k}$. Therefore, traditional SE is not a meaningful parameter to measure the performance of an RFID system. To mitigate the different slots’ duration effect, the metric Time_SE is introduced in [27] $Time\_SE=c_{s}/\left[c_{total}+\left(\beta -1\right)c_{i}\right]$, where $c_{total}$ = $c_{i}$ + $c_{s}$ + $c_{k}$ and β = $T_{i}/T_{k}$. This metric considers different durations for $T_{k}$ and $T_{i}$, but it assumes $T_{s}$ = $T_{k}$ and does not include the time overhead information.

In order to provide an accurate evaluation of an RFID system, this work focuses on the metric *E*/*n*, defined as the energy per tag identified consumed by the reader in one inventory round. This metric considers different durations for $T_{i}$, $T_{s}$ and $T_{k}$.

The energy consumed by the reader during the identification of a whole set of tags, defined as *E*, is modeled in [4]. The authors present an energy consumption model where *E* depends on the power required by the reader to transmit and receive information to and from the tags. During the identification process, the reader transmits a set of commands and a Continuous Wave (CW) to power up passive tags, with power $P_{t_{x}}$. To receive the data from the tags, the reader needs an extra power $P_{r_{x}}$. Defining $E_{t}$/*n* and $E_{r}$/*n* as the energy per tag identified consumed by the reader during the transmitting and receiving states in one inventory round, one obtains:

(1) $$\frac{E}{n}=\frac{E_{t}}{n}+\frac{E_{r}}{n}$$

where:

(2) $$E_{t}=P_{t_{x}}\left(c_{s}T_{s}+c_{k}T_{k}+c_{i}T_{i}\right)$$

and:

(3) $$E_{r}=P_{r_{x}}\;\left[c_{s}\left(T_{RN16}+T_{EPC}\right)+c_{k}T_{RN16}\right]$$

The durations of the slots, $T_{i}$, $T_{s}$ and $T_{k}$, are set according to the standard:

(4) $$T_{i}=T_{1}+T_{3}+T_{command},$$

(5) $$T_{s}=2T_{1}+T_{RN16}+2T_{2}+T_{ACK}+T_{EPC}+T_{command},$$

and:

(6) $$T_{k}=T_{1}+T_{RN16}+T_{2}+T_{command}$$

where $T_{command}$ refers to the duration of the reader transmitted command Qc, QA or QR, referred to as $T_{Qc}$, $T_{QA}$ and $T_{QR}$, respectively. In every frame, the reader will transmit just one QA or Qc in the first slot, and in the rest of slots, it will transmit QR commands. Assuming a frame with sufficiently large *L*, $T_{command}$ = $T_{QR}$ is applied in (4)–(6) when one frame is analyzed.

The parameters $T_{Qc}$, $T_{QA}$ and $T_{QR}$ are calculated as the reader-to-tag synchronization time $T_{FSync_{RT}}$ or $T_{Preamble_{RT}}$, defined in [8] plus the length of each parameter divided by the reader data rate $DR_{r}$, calculated as $DR_{r}$ = $1/(\left(T_{data_{0}}+T_{data_{1}}\right)/2)$, where $T_{data_{0}}$ = Tari and $T_{data_{1}}$ = 1.5·Tari. Tari represents the reference time interval for a data-0 transmission. Thus, $T_{Qc}$ = $T_{FSync_{RT}}$ + 22 bits/$DR_{r}$, $T_{QA}$ = $T_{Preamble_{RT}}$ + 9 bits/$DR_{r}$ and $T_{QR}$ = $T_{Preamble_{RT}}$ + 4 bits/$DR_{r}$

The parameter $T_{RN16}$ and $T_{EPC}$ refer to the time the tag employs to transmit RN16 and its EPC, respectively. They are calculated as the tag-to-reader synchronization time $T_{Preamble_{TR}}$ plus the length of each parameter divided by the tag data rate $DR_{t}$, calculated as $DR_{t}$ = BLF/M. The parameter BLF refers to the backscatter-link frequency. Thus, $T_{RN16}$ = $T_{Preamble_{TR}}$ + 17 bits/$DR_{t}$ and $T_{EPC}$ = $T_{Preamble_{TR}}$ + 129 bits/$DR_{t}$. Finally, $T_{ACK}$ corresponds to the duration of the reader command ACK, and it is obtained as $T_{ACK}$ = $T_{Preamble_{RT}}$ + 18 bits/$DR_{r}$. Table 1 summarizes the calculation of the reader and tag messages’ durations.

In the next section, the energy per tag identified consumed by the reader in one frame is analyzed, and the optimal *L* is derived to minimize the reader energy consumption in an RFID identification process.

### Frame Size Calculation to Minimize the Reader Energy Consumption

Defining E(n,L)/$c_{s}\left(n,L\right)$ as the energy per tag identified consumed by the reader in one frame, one obtains:

(7) $$\frac{E(n,L)}{c_{s}\left(n,L\right)}=\frac{E_{t}\left(n,L\right)}{c_{s}\left(n,L\right)}+\frac{E_{r}\left(n,L\right)}{c_{s}\left(n,L\right)}$$

where:

(8) $$\frac{E_{t}\left(n,L\right)}{c_{s}\left(n,L\right)}=\frac{P_{t_{x}}\;\left[\left(c_{s}\left(n,L\right)T_{s}+c_{k}\left(n,L\right)T_{k}+c_{i}\left(n,L\right)T_{i}\right)\right]}{c_{s}\left(n,L\right)}$$

and:

(9) $$\frac{E_{r}\left(n,L\right)}{c_{s}\left(n,L\right)}=\frac{P_{r_{x}}\;\left[c_{s}\left(n,L\right)\left(T_{RN16}+T_{EPC}\right)+c_{k}\left(n,L\right)T_{RN16}\right]}{c_{s}\left(n,L\right)}$$

where $c_{s}\left(n,L\right)$, $c_{k}\left(n,L\right)$ and $c_{i}\left(n,L\right)$ represent the expected value of the number of single, collision and idle slots in a frame, respectively. From (8) and (9), it follows that E(n,L)/$c_{s}\left(n,L\right)$ is mainly influenced by the number of each type of slot and their duration. On the one hand, $T_{i}$, $T_{s}$ and $T_{k}$ are fixed for a particular RFID system, and they remain constant for one frame. On the other hand, $c_{i}\left(n,L\right)$, $c_{s}\left(n,L\right)$ and $c_{k}\left(n,L\right)$ strictly depend on the anti-collision protocol employed to identify the tag set and, particularly, on the strategy it uses to update *L*.

In order to perform the analysis that derives the optimal *L* to minimize $E\left(n,L\right)/c_{s}\left(n,L\right)$, a system model with one reader and *n* tags is defined. The probability that *b* tags among *n* occupy a slot within a frame of size *L* can be approximated by a binomial distribution $P_{b}\left(n,L\right)$ [28]:

(10) Pb(n,L)=nb1Lb1−1Ln−b.

If *L* is assumed sufficiently large, the tags distribution can be approximated by a Poisson distribution with mean ρ.

(11) $$\rho =\frac{n}{L}.$$

When b=0 in (10), $c_{i}\left(n,L\right)$ can be approximated by:

(12) $$c_{i}\left(n,L\right)=Lp_{i}\left(n,L\right)=L{\left(1-\frac{1}{L}\right)}^{n}\approx Le^{-\rho }.$$

When b=1 in (10), $c_{s}\left(n,L\right)$ can be approximated by:

(13) $$c_{s}\left(n,L\right)=Lp_{s}\left(n,L\right)=n{\left(1-\frac{1}{L}\right)}^{n-1}\approx L\rho \left(\frac{n/\rho }{n/\rho -1}\right)e^{-\rho }.$$

Then, $c_{k}\left(n,L\right)$ can be approximated by:

(14) $$c_{k}\left(n,L\right)=L\cdot p_{k}\left(n,L\right)=L\left(1-p_{0}-p_{1}\right).$$

Substituting (12)–(14) into (8) and (9) and applying $\frac{n/\rho }{n/\rho -1}$ ≈ 1, the following expressions are obtained:

(15) $$\frac{E_{t}\left(n,L\right)}{c_{s}}\approx \;\frac{P_{t_{x}}\left[T_{s}\rho e^{-\rho }+T_{i}e^{-\rho }+T_{k}\left(1-\left(1+\rho \right)e^{-\rho }\right)\right]}{\rho e^{-\rho }}$$

and:

(16) $$\frac{E_{r}\left(n,L\right)}{c_{s}}\approx \;P_{r_{x}}\frac{T_{RN16}\;\rho e^{-\rho }-T_{EPC}\;e^{-\rho }+T_{EPC}}{\rho e^{-\rho }}.$$

Next, computing the derivative of $E\left(n,L\right)/c_{s}\left(n,L\right)$ in (7) with respect to ρ yields:

(17) $$\frac{d}{d\rho }\frac{E(n,L)}{c_{s}\left(n,L\right)}=\frac{d}{d\rho }\frac{E_{t}\left(n,L\right)}{c_{s}\left(n,L\right)}+\frac{d}{d\rho }\frac{E_{r}\left(n,L\right)}{c_{s}\left(n,L\right)}=P_{t_{x}}\frac{T_{k}\left[e^{\rho }\left(\rho -1\right)+1\right]-T_{i}}{\rho^{2}}+P_{r_{X}}\frac{T_{RN16}\;e^{\rho }\left(\rho -1\right)+T_{EPC}}{\rho^{2}}$$

and posing $\frac{d}{d\rho }\frac{E(n,L)}{c_{s}\left(n,L\right)}$ = 0 yields the following equation:

(18) $$\rho e^{\rho }-e^{\rho }+1=\frac{P_{r_{x}}T_{i}}{P_{t_{x}}T_{k}+P_{r_{x}}T_{EPC}}.$$

Solving (18), the value of ρ that minimizes $E\left(n,L\right)/c_{s}\left(\rho \right)$ is obtained:

(19) $$\rho =1+W\left[\frac{P_{t_{x}}\left(T_{i}-T_{k}\right)-P_{r_{x}}T_{EPC}}{e(P_{t_{x}}T_{k}+P_{r_{x}}T_{EPC})}\right]$$

where W(x) represents the Lambert W-function.

Then, according to (11), the frame size that minimizes $E\left(n,L\right)/c_{s}\left(n,L\right)$ is obtained as *L* = *n*/ρ. Following the EPC C1G2 constraints, the *L* value must be a power of two. Thus, *Q* = $round(log_{2}\left(n/\rho \right))$ and:

(20) $$L=2^{round[log_{2}\left(n/\rho \right)]}.$$

## 4. RFID Anti-Collision Protocol Energy-Aware Slotted Aloha

In this section, the analysis of the energy-aware Aloha frame presented in Section 3 is applied to a DFSA anti-collision protocol, resulting in the Energy-Aware Slotted Aloha (EASA) protocol. EASA is based on EPC C1G2, regarding the reader and the tag operation. The pseudocode of EASA is presented in Algorithm 1. First the operation of the reader is presented, then the operation of the tag. The variable slot_index represents the reader’s internal counter, which keeps track of the present slot in the current frame.
**Algorithm 1** Pseudocode of EASA. First, the operation of the reader is presented, then the operation of the tag.**Reader Operation**1:Initialization: slot_index = 1, $L=2^{Q}$2:calculate ρ by solving (19) 3:broadcast Qc 4:**while** 1 **do**5: read slot and update $c_{i},c_{s},c_{k}$
6: **if** slot_index = *L* **then**7:  $\hat{n}=MMSE$($c_{i},c_{s},c_{k}$) 8:  $Q=log_{2}\left[(\hat{n}-c_{s})/\rho \right]$, $L=2^{round(Q)}$9:  broadcast QA10: **else**11:  slot_index = slot_index +1 12:  broadcast QR 13: **end if**14:**end while****Tag Operation**1:**while** energized by the reader **do**2: receive reader’s commands 3: **if** QA or Qc **then**4:  Generate SC ∈ [0,L−1]5: **else**6:  **if** QR **then**7:   SC = SC−18:  **end if**9: **end if**10: **if** SC = 0 **then**11:  transmit RN16
12:  **if** ACK **then**13:   transmit EPC
14:  **end if**15: **end if**16:**end while**

First, the reader sets the value of ρ by solving (19). The value of ρ is obtained just once at the beginning of the inventory round, according to the RFID system parameters $P_{t_{x}}$, $P_{r_{x}}$, $T_{i}$, $T_{k}$ and $T_{EPC}$. These parameters define the particular RFID system used. Then, the initial *L* to begin the identification process is obtained with (20), and the reader starts the identification procedure by broadcasting Qc, specifying the initial *Q* value of the tags. Each tag selects a slot in the frame to transmit its EPC. The reader continues the identification process analyzing each slot of the frame, updating the variables $c_{i}$, $c_{s}$ and $c_{k}$ according to the tags’ responses:

- Only one tag response is detected: $c_{s}$ = $c_{s}$ + 1.
- Two ore more tags response are detected: $c_{k}$ = $c_{k}$ + 1.
- No tag response is detected: $c_{i}$ = $c_{i}$ + 1.

Then, the reader broadcasts QR to go from one slot to the next. When the reader reaches the last slot of the frame, the remaining tag population size is estimated with a traditional Mean Minimum Square Error (MMSE) estimator [28]:

(21) $$\begin{array}{cc} \hat{n}=\underset{n}{min} & {∥\left(\begin{array}{c} c_{i}\left(n,L\right)\\ c_{s}\left(n,L\right)\\ c_{k}\left(n,L\right) \end{array}\right)-\left(\begin{array}{c} c_{i}\\ c_{s}\\ c_{k} \end{array}\right)∥}^{2}=\underset{n}{min}\left\{{\left[c_{i}\left(n,L\right)-c_{i}\right]}^{2}+{\left[c_{s}\left(n,L\right)-c_{s}\right]}^{2}+{\left[c_{k}\left(n,L\right)-c_{k}\right]}^{2}\right\}. \end{array}$$

This estimator compares the expected value of the number of idle, single and collision slots at the end of the frame ($c_{i}\left(n,L\right)$, $c_{s}\left(n,L\right)$ and $c_{k}\left(n,L\right)$, obtained with (12), (13) and (14)), with the observed numbers ($c_{i}$, $c_{s}$ and $c_{k}$). Then, $\hat{n}$ is obtained as the value of *n*, which minimizes the mean square error of the expected and observed values. Finally, the frame size is updated with (20), and a new frame is started by broadcasting QA, specifying the new *L*. The tag operation follows the EPC C1G2 standard behavior (see Section 2.1). Therefore, EASA is compatible with commercial RFID tags.

EASA presents an energy-aware frame, because this protocol sets the frame size according to ρ and $\hat{n}$ so that *E*/*n* is minimized. Besides, if the timing parameters of the RFID system vary, EASA adapts to these changes, obtaining a new solution for ρ and updating *L* accordingly. Furthermore, EASA can be physically implemented in a real system and commercial tags, because EASA is based on EPC C1G2.

## 5. Energy Evaluation

This section evaluates the performance of EASA regarding the energy per tag identified consumed by the reader in one inventory round E/n and compares it with the anti-collision protocols of the state of the art presented in Section 2.2: Eom [21], ILCM-FbF [7], ILCM-SbS [22], Chen14 [23], Chen16 [24], SSA [25] and DSSA [25]. Physical-layer effects are not considered here, assuming a non-impaired channel and no capture effect. Note that these assumptions are extensively used for the analysis of known anti-collision protocols whose analysis focuses on the media access control layer [4,21,24,25]. Simulation results were obtained with MATLAB R2017b. A scenario with one reader and a varying number of tags is evaluated, where the tags are uniformly distributed. The simulation responses have been averaged over 1000 iterations for accuracy in the results. Timing parameters are set according to Table 2. Before proceeding with the performance evaluation, some implementation details must be taken into consideration:

- To evaluate the anti-collision protocols’ performance with *n*, the tag set sizes considered are *N* = [16,32,64,128,256,512,1024,2048] and *n* ∈ *N*.
- *L* values are limited to a power of two, following the EPC C1G2 specifications.
- The initial *L* is set to 16 (*Q* = 4), following the EPC C1G2 recommendation.
- The length of the EPC is set to *k* = 128.

**Figure 2:**
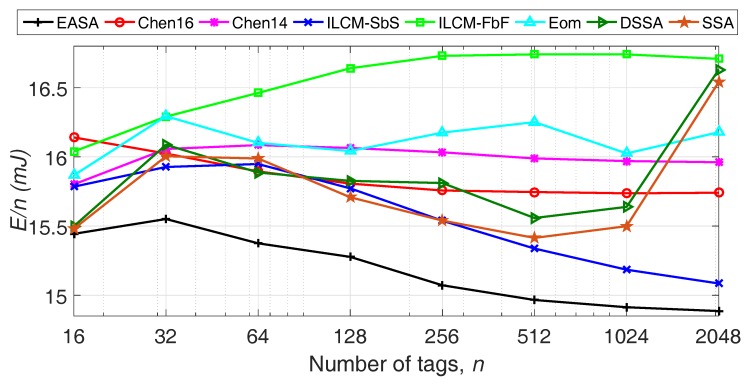
Evaluation of the energy consumption per tag in one inventory round, varying *n* from 16–2048, with $P_{t_{x}}$ = 825 mW and $P_{r_{x}}$ = 125 mW. EASA, Energy-Aware Slotted Aloha; ILCM-SbS, Improved Linearized Combinational Model with Slot by Slot; FbF, Frame by Frame; DSSA, Dynamic Segment-by-Segment Aloha.

Figure 2 shows that EASA clearly improves E/n for all *n* evaluated, with an average of 15 mJ consumed by the reader per tag identified. Overall, EASA presents a 6% average reduction in E/n compared to ILCM-SbS, the protocol with the second lowest E/n. Additionally, for all the protocols in the comparison, low variations of E/n with *n* are obtained, presenting a quasi-constant behavior for all the range of *n* evaluated. The strategies Chen14, SSA and DSSA present an increasing peak in E/n around *n* = 1024 and *n* = 2048, because they limit *L* to 1024 in both situations. From this figure, it can be concluded that the solution of ρ obtained for one frame results in the lowest energy consumed by the reader in one inventory round.

Next, all the algorithms presented in Section 2.2 are evaluated in terms of the number of slots and the reader and tag bits, in order to provide a deeper insight into the E/n results. Results are averaged for *N*.

**Figure 3:**
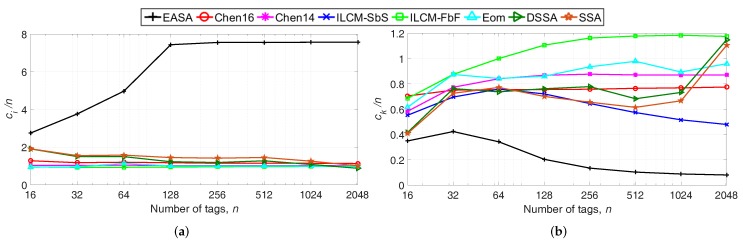
(**a**) Total number of idle slots per tag $c_{i}/n$ and (**b**) total number of collision slots per tag $c_{k}/n$, varying *n* from 16–2048.

Figure 3 shows the evaluation results of the number of slots. The proposed protocol presents the highest $c_{i}$, with around 3–7 idle slots per tag, while the alternative protocols show around 1–2 idle slots per tag. All protocols in the comparison update *L* with a power of two value close to *n*, except for EASA and Chen16. EASA updates *L* with (20), where ρ = 4.13, and Chen16 updates *L* with *L* =y$\hat{n}$, where *y* = 1.2. Because EASA generates frames with a size around 4.13-times the estimated number of tags (scaled to a power of two value), it generates a higher number of slots than the alternative protocols, resulting in a higher number of idle slots. However, idle slots are the shortest of the three types, having a low impact on the total identification time. In particular, according to Table 2, an idle slot is around 27-times shorter than a collision slot. In relation to $c_{k}$, the proposed protocol achieves the lowest value of 0.1–0.4 collision slots per tag.

Recovering the results obtained in Figure 2, it is noticed that the reduction in the number of collision slots per tag of EASA results in a lower E/n. That is, the increase in the number of idle slots is compensated with a decrease in the number of collision slots.

**Figure 4:**
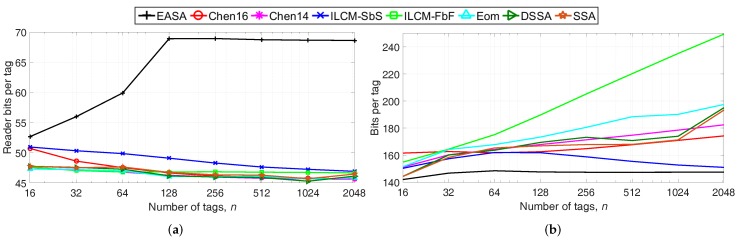
(**a**) Reader bits per tag and (**b**) bits per tag to identify *n* tags, varying *n* from 16–2048.

Next, the total number of reader transmitted bits per tag and the average number of bits transmitted by one tag are evaluated, because these metrics also influence E/n. Results are shown in Figure 4a,b.

Regarding Figure 4a, EASA presents the highest number of total reader bits per tag because of the higher number of $c_{i}$/*n* generated. Despite the higher values of the number of reader bits of EASA, the proposed protocol achieves an improved performance in terms of E/n. This occurs because EASA greatly reduces the total number of collision slots, reducing the total reader and tag waiting periods. These waiting periods are represented by the link timing parameters $T_{1}$, $T_{2}$ and $T_{3}$, as defined in Section 2. During these periods, the reader and the tags do not transmit any bit, but the reader consumes energy from its battery by transmitting the CW. Using the values of Table 2, the total waiting time of a collision slot is defined by $T_{1}$ + $T_{2}$ = 162.50 μs, while the total waiting time of an idle slot is $T_{1}$ + $T_{3}$ = 96.25 μs. Therefore, the waiting time of a collision slot is about 1.7-times higher than that of an idle slot. Overall, it can be concluded that EASA results in energy savings despite the higher number of reader bits per tag, because it highly reduces the number of collision slots and the waiting time periods.

Regarding Figure 4b, EASA presents the lowest number of bits per tag compared the rest of the strategies, with an average of 146 bits. Because EASA generates fewer $c_{k}/n$ (see Figure 3b), tags suffer fewer collisions, and therefore, they transmit fewer bits.

### 5.1. Effect of $P_{t_{x}}$ and $P_{r_{x}}$ on E/n

Next, the total energy consumption for *n* tags’ identification is evaluated when $P_{t_{x}}$ and $P_{r_{x}}$ are varied for two different tag set sizes (*n* = 64 and 1024). Firstly, E/n is evaluated when $P_{r_{x}}$ is fixed to 125 mW, and $P_{t_{x}}$ takes two values, 125 and 825 mW. Next, E/n is evaluated when $P_{t_{x}}$ is fixed to 825 mW, and $P_{r_{x}}$ takes the values 25 and 800 mW. These values of $P_{t_{x}}$ and $P_{r_{x}}$ have been selected considering that a typical commercial RFID reader, by regulation, provides a maximum output power of 1 W. The rest of the parameters are set according to Table 2. Simulation results are shown in Table 3. When $P_{r_{x}}$ is set to 125 mW, all the protocols in the comparison show a similar behavior, presenting an increasing E/n with increasing *n* and increasing $P_{t_{x}}$. When $P_{t_{x}}$ is set to 825 mW, E/n increases with increasing *n*, but it hardly varies for the two $P_{r_{x}}$ values analyzed. In conclusion, for all the protocols analyzed, the reader transmitting power $P_{t_{x}}$ greatly affects the energy per tag identified consumed by the reader in one inventory round, and the higher $P_{t_{x}}$ for a particular $P_{r_{x}}$, the higher E/n. Furthermore, it can be seen that the protocols with the lowest number of slots (see Figure 3) and bits per tag (see Figure 4) present the lowest energy consumption (see Figure 2). Therefore, these two parameters are key regarding the energy efficiency of an anti-collision protocol.

EASA presents the lowest E/n for all the combinations of $P_{t_{x}}$ and $P_{r_{x}}$ evaluated. This occurs because the value of ρ is calculated as a function of these parameters (see (19)). Therefore, EASA presents an energy-aware behavior, because this protocol adapts to different values of $P_{t_{x}}$ and $P_{r_{x}}$, lowering the overall *E*/*n* in relation to the alternative protocols.

### 5.2. Effect of Tari and BLF on E/n

The results shown in Figure 2 correspond to a specific RFID system, with a particular set of timing parameters, defined in Table 2. This work assumes that the two key parameters that define an RFID system are Tari and BLF, because most of the configuration parameters are obtained as a function of these two. Next, the protocols’ performance is evaluated in terms of E/n for different RFID systems with varying Tari and BLF. The two parameters are varied from the whole range of values allowed by EPC C1G2 [8]. The values of $P_{t_{x}}$ and $P_{r_{x}}$ used are set according to Table 2.

The different values of Tari and BLF greatly affect the performance of EASA, because they result in different values of ρ to update the frame size *L*. As was shown in Section 3, the value of ρ is obtained as a function of $T_{i}$, $T_{k}$ and $T_{EPC}$ (see (19), which ultimately depends on Tari and BLF. Therefore, the value of ρ also depends on Tari and BLF

Firstly, Tari is set to 25 μs, while BLF is varied from 40–640 kbps. Then, BLF is set to 40 kbps, and Tari is varied from the minimum (6.25 μs) to the maximum (25 μs) value allowed by EPC C1G2. As a result, 1/ρ varies from 1.36–4.13. The values of $T_{1}$, $T_{2}$ and $T_{3}$ are also affected because they are set as a function of Tari and BLF. The evaluated results are averaged for *N* and shown in Table 4.

Overall, *E*/*n* decreases with increasing BLF for a particular Tari for all the protocols evaluated. It is important to note that a higher BLF involves a faster tag (higher data rate $DR_{t}$). Therefore, if $DR_{t}$ increases, the reader consumes a lower amount of receiving power $E_{r}$, reducing the overall *E*/*n*.

EASA presents the lowest *E*/*n* when 1/ρ ≥ 2.66. Therefore, there is evidence that EASA presents an energy-aware frame. Furthermore, the higher the value of 1/ρ, the more notable the improvement in *E*/*n* of the proposed protocol in relation to the rest of the strategies. This occurs because the frame size of EASA increases with increasing 1/ρ (see (20)), decreasing the probability of collisions. As 1/ρ decreases, the performance of EASA deteriorates. In this situation, the probability of collision in EASA is higher than in the previous situation (*L* is smaller for the same *n*). As a result, the reader consumes a higher amount of transmitting and receiving power $E_{r}$ and $E_{t}$, increasing the overall *E*/*n* of EASA.

### 5.3. Analysis of Communication Channel Effects on the Battery Lifetime

An ideal communication channel has been considered in the previous analysis, where there are no communication errors between the reader and the tags, and tags are uniformly distributed inside the reader interrogation zone. However, the capture effect is very common in passive RFID systems [29]. This phenomenon occurs when the reader successfully resolves one tag reply in a collided slot. A different effect is the detection error [30], which means that a single tag response is detected as idle, due to fading or interference. As a result, re-transmissions are required in subsequent slots.

These two effects will influence the protocols’ performance in terms of the metric E/n. As a result, the reader battery lifetime will be also affected. This section evaluates the protocol’s performance in terms of the reader’s battery duration under the capture effect and the detection error. For this purpose, an RFID system with *n* passive tags and one battery-operated reader is considered. The reader operates from a lithium rechargeable battery, which has 0.48 kJ of energy [31]. The reader is assumed to transmit until all *n* tags are read and is not affected by its orientation to the tags.

Next, the percentage of the reader energy consumed from the battery to identify *n* = 1024 tags with respect to the total battery capacity is measured when the capture effect $P_{c}$ and the probability of a detection error $P_{d}$ are present in the communication channel. First, *E* (J) is evaluated for *n* = 1024 for all the protocols. Then, the percentage is obtained considering that 0.48 kJ represents 100% of the battery capacity. The timing parameters of Section 5.1 are used. Simulation results are shown in Table 5.

On the one hand, for a fixed $P_{d}$, the reader battery consumption decreases with increasing $P_{c}$ for all the protocols in the comparison because fewer collided slots and more single slots occur. On the other hand, for a fixed $P_{c}$, the battery consumption increases with increasing $P_{d}$, because a higher number of total slots is required to complete one inventory round.

Table 5 shows that the improvement of EASA in relation to the rest of the protocols in the comparison becomes less significant with increasing $P_{c}$. The capture effect leads to collision slots becoming single slots. Therefore, a high $P_{c}$ will be favorable to protocols with a high number of collision slots. Because EASA achieves the lowest number of collision slots (see Figure 3b), $P_{c}$ has a smaller impact on EASA performance compared to the alternative protocols. This behavior is more notable for $P_{d}$ = $P_{c}$ = 0.2, where EASA and ILCM show the same percentage.

Assuming a reader with a lithium rechargeable battery of 0.48 kJ of energy, the reader will be capable of reading up to 32,201 tags with the EASA protocol (performing 31.45 identification cycles of 1024 tags) before the battery is empty (in the scenario with $P_{d}$ = $P_{c}$ = 0). In contrast, if the reader uses ILCM-FbF (the protocol with the highest *E*), the number of tags read is reduced to 28,683 tags, which represents 3517.8 fewer tags than in the case of using EASA.

The results of this section show that the battery consumption of a mobile RFID reader that is working in an communication channel with the capture effect and detection error will be minimized if the reader employs EASA. Therefore, EASA provides a longer battery lifetime for the reader than the alternative protocols.

## 6. Conclusions and Future Work

A novel RFID anti-collision protocol based on the current standard EPC-global Class-1 Generation-2 has been presented to decrease the energy per tag consumed by the reader E/n. EASA presents an energy-aware frame, because this protocol sets the frame size according to the parameters ρ and $\hat{n}$ so that E/n is minimized.

The metric E/n has been thoroughly studied, analyzing the main factor that affects it: the frame size *L* update strategy. From this study, it has been concluded that in order to decrease E/n, *L* must be 1/ρ-times the estimated number of tags, where ρ is set according to the timing and power parameters of the RFID system $T_{i}$, $T_{k}$, $T_{EPC}$, $P_{t_{x}}$ and $P_{r_{x}}$.

The proposed protocol was compared with several protocols of the state of the art in relation to E/n and the number of slots and transmitted bits. Simulation results showed that EASA, with an average 15 mJ per tag consumed by the reader, achieves a 6% average improvement in E/n in relation to the strategies of the comparison. Therefore, EASA is a suitable candidate where energy-efficiency is sought in passive RFID.

Several works in the literature present an SDR-RFID system [32,33,34] to evaluate the performance of the slot counter protocol (used in EPC C1G2) in a real scenario. In these systems, it would be possible to analyze the capture effect and the detection error in a real scenario. Because EASA is based on EPC C1G2, it can be physically implemented with an SDR-RFID system and commercial tags. Although this implementation is out of the scope of this work, it is proposed as future work.

## Figures and Tables

**Table 1:** Main EPC C1G2 timing parameters’ calculation.

Parameter	Description	Calculation
$T_{data_{0}}$	Duration of a reader data-0	Tari
$T_{data_{1}}$	Duration of a reader data-1	1.5·Tari
$T_{Preamble_{RT}}$	Duration of R-T Preamble	$T_{del}+T_{data_{0}}+RTcal+TRcal$
$T_{FSync_{RT}}$	Duration of Frame Sync.	$T_{del}+T_{data_{0}}+RTcal$
$T_{Preamble_{TR}}$	Duration of T-R	7/DRt
DRr	Reader data rate	$1/(\left(T_{data_{0}}+T_{data_{1}}\right)/2)$
DRt	Tag data rate	BLF/M
$T_{Q_{c}}$	Duration of a Qc	$T_{FSync_{RT}}$ + 22/$DR_{r}$
$T_{QA}$	Duration of a QA	$T_{Preamble_{RT}}$ + 9/$DR_{r}$
$T_{QR}$	Duration of a QR	$T_{Preamble_{RT}}$ + 4/$DR_{r}$
$T_{ACK}$	Duration of a ACK	$T_{FSync_{RT}}$ + 24/$DR_{r}$
$T_{RN16}$	Duration of tag RN16	23/DRt
$T_{EPC}$	Duration of tag EPC	135/DRt

**Table 2:** Simulation parameters according to EPC C1G2 [8].

Parameter	Value	Parameter	Value
Tari	6.25μs	BLF	40 kbps
$T_{Preamble_{RT}}$	234.38μs	$T_{1}$	87.5μs
$T_{Preamble_{TR}}$	700.00μs	$T_{2}$	75μs
$T_{FSync_{RT}}$	34.38μs	$T_{3}$	8.75μs
$DR_{r}$	128 kbps	$DR_{t}$	10 kbps
$T_{QA}$	104.69μs	$T_{QR}$	65.63μs
$T_{Q_{C}}$	406.25μs	$T_{QE}$	65.63μs

**Table 3:** Energy consumption per tag *E*/*n* (mJ) evaluation varying $P_{t_{x}}$ and $P_{r_{x}}$ for *n* = 64 and *n* = 1024. Each combination of $P_{t_{x}}$ and $P_{r_{x}}$ results in a different ρ value for EASA. Quantities in bold represent the best results among the strategies in the comparison.

	$P_{r_{x}}=125$ mW				$P_{r_{x}}=25$ mW		$P_{r_{x}}=800$ mW	
	$P_{t_{x}}=125$ **mW**		**mW**		$P_{t_{x}}=825$ **mW**			
1/ρ	**7.41**		**4.13**		**3.39**		**7.32**	
**Protocol**	n **= 64**	n **= 1024**	n **= 64**	n **= 1024**	n **= 64**	n **= 1024**	n **= 64**	n **= 1024**
EASA	**2.63**	**2.44**	**15.32**	**14.91**	**15.10**	**238.04**	**17.20**	**257.10**
Chen16	2.76	2.60	15.86	15.73	15.56	248.63	18.12	273.58
Chen14	2.81	2.66	16.10	15.98	15.75	279.68	18.51	279.68
ILCM-SbS	2.77	**2.44**	15.84	15.19	15.60	240.69	18.17	257.88
ILCM-FbF	2.92	2.85	16.46	16.74	16.10	262.95	19.09	300.00
Eom	2.83	2.67	16.19	16.03	15.80	252.84	18.52	281.47
DSSA	2.77	2.57	16.00	15.64	15.66	247.32	18.16	271.18
SSA	2.76	2.53	15.88	15.50	15.63	245.10	18.12	266.85

**Table 4:** Study of the effect of Tari and BLF on E/n (mJ). Results are averaged for *N*. Tari and BLF are varied from the maximum to the minimum values allowed by EPC C1G, resulting in different ρ values for EASA. $P_{t_{x}}$ and $P_{r_{x}}$ are set to 825 mW and 125 mW, respectively. Quantities in bold represent the best results among the protocols in the comparison.

Tari (μs)	25	25	25	25	25	25	16	11.43	8.89	7.27	6.25
BLF (kbps)	640	320	213.3	160	64	40	40	40	40	40	40
1/ρ	**1.36**	**1.61**	**1.81**	**1.98**	**2.66**	**3.07**	**3.44**	**3.71**	**3.90**	**4.03**	**4.13**
EASA	2.13	3.10	4.12	5.04	**10.53**	**16.40**	**15.87**	**15.56**	**15.37**	**15.24**	**15.17**
Chen16	2.12	**3.09**	**4.06**	**5.03**	10.85	16.67	16.28	16.07	15.94	15.88	15.84
Chen14	**2.06**	3.10	4.07	5.05	10.93	16.81	16.42	16.23	16.11	16.05	16.00
ILCM-SbS	2.16	3.12	4.08	**5.03**	10.73	16.43	16.02	15.84	15.72	15.63	15.56
ILCM-FbF	2.17	3.18	4.19	5.20	11.28	17.37	16.98	16.77	16.67	16.58	16.54
Eom	2.11	3.11	4.09	5.08	11.02	16.94	16.55	16.35	16.23	16.15	16.12
DSSA	2.13	3.10	4.08	5.05	10.88	16.73	16.31	16.12	15.98	15.92	15.87
SSA	2.15	3.10	4.08	5.04	10.83	16.62	16.23	16.01	15.90	15.80	15.75

**Table 5:** Percentage (%) of the reader battery consumed to identify *n* = 1024 tags. The reader uses a lithium battery, which has 0.48 kJ of energy [31]. The protocols are evaluated in terms of the probability of the capture effect $P_{c}$ and the probability of a detection error $P_{d}$. The timing parameters of Section 5.1 are used. Quantities in bold represent the best results among the protocols in the comparison.

$P_{d}$	0	0	0	0.2	0.2	0.2
$P_{c}$	**0**	**0.1**	**0.2**	**0**	**0.1**	**0.2**
EASA	**3.18**	**3.18**	**3.17**	**3.22**	**3.21**	**3.20**
Chen16	3.36	3.30	3.25	3.33	3.28	3.25
Chen14	3.41	3.35	3.30	3.44	3.39	3.35
ILCM-SbS	3.24	3.22	3.19	3.25	3.22	**3.20**
ILCM-FbF	3.57	3.51	3.46	3.61	3.50	3.44
Eom	3.42	3.39	3.40	3.43	3.44	3.37
DSSA	3.31	3.26	3.24	3.24	3.24	3.23
SSA	3.34	3.31	3.33	3.32	3.33	3.33
